# Frequent Gene Amplification Predicts Poor Prognosis in Gastric Cancer

**DOI:** 10.3390/ijms13044714

**Published:** 2012-04-13

**Authors:** Jing Shi, Demao Yao, Wei Liu, Na Wang, Hongjun Lv, Nongyue He, Bingyin Shi, Peng Hou, Meiju Ji

**Affiliations:** 1 Department of Endocrinology, The First Affiliated Hospital of Xi’an Jiaotong University School of Medicine, Xi’an 710061, China; E-Mails: sj1840@163.com (J.S.); liulw212@126.com (W.L.); wangna526455178@163.com (N.W.); njdxbjdx@163.com (H.L.); shibingy@126.com (B.S.); 2 Department of Surgery, The First Affiliated Hospital of Xi’an Jiaotong University School of Medicine, Xi’an 710061, China; E-Mail: yaodemao@yahoo.com.cn; 3 State Key Laboratory of Bioelectronics, Southeast University, Nanjing 210096, China; E-Mail: nyhe1958@163.com; 4 Center for Translational Medicine, The First Affiliated Hospital of Xi’an Jiaotong University School of Medicine, Xi’an 710061, China

**Keywords:** gastric cancer, oncogenes, gene amplification, poor prognosis

## Abstract

Gastric cancer is one of the most common malignancies worldwide. However, genetic alterations leading to this disease are largely unknown. Gene amplification is one of the most frequent genetic alterations, which is believed to play a major role in the development and progression of gastric cancer. In the present study, we identified three frequently amplified genes from 30 candidate genes using real-time quantitative PCR method, including *ERBB4*, *C-MET* and *CD44*, and further explored their association with clinicopathological characteristics and poor survival in a cohort of gastric cancers. Our data showed amplification of these genes was significantly associated with certain clinicopathological characteristics, particularly tumor differentiation and cancer-related death. More importantly, amplification of these genes was significantly related to worse survival, suggesting that these amplified genes may be significant predictors of poor prognosis and potential therapeutic targets in gastric cancer. Targeting these genes may thus provide new possibilities in the treatment of gastric cancer.

## 1. Introduction

Gastric cancer is one of the most common malignancies and remains the second leading cause of cancer-related death worldwide [1]. Over 70% of new cases and deaths occur in developing countries. The highest incidence rates are in Eastern Asia, Eastern Europe and South America, particularly China [2]. Although recent diagnostic and therapeutic advances have gradually improved clinical outcome of the patients with early gastric cancer, unfortunately, gastric cancer is usually diagnosed at an advanced stage and the prognosis is still poor [3], reflecting limited advances in our understanding of the pathogenesis of this disease. Thus, a better understanding of the molecular mechanisms and genetic alterations of gastric cancer may lead to new diagnostic and therapeutic strategies to this disease.

Gene amplification is one of the most frequent genomic alterations found in human cancers, including gastric cancer [4–6]. Increased gene dosage by this genetic event is a common mechanism for oncogene overexpression during tumorigenesis [7]. Generally, the amplified genes do not undergo additional damage by mutations, indicating that it is the enhanced levels of a wide-type protein that contributes to tumorigenesis [7]. Like other types of genetic alterations, gene amplification also reflects the genetic instability of the tumor cells, and may confer diagnostic, prognostic, or therapeutic information for patient management [8].

Using real-time quantitative PCR method, we identified three frequently amplified genes from 30 candidate genes, including *ERBB4*, *C-MET* and *CD44* genes, in 30 pairs of gastric cancer and normal gastric tissues, and further demonstrated that aberrant amplification of these genes significantly affected the clinical outcome in a cohort of clinically well-characterized gastric cancers.

## 2. Results

### 2.1. Highly Frequent Amplification of ERBB4, C-MET and CD44 in Gastric Cancer

**Figure 1:**
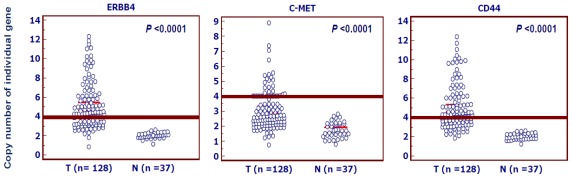
The copy number of *ERBB4*, *C-MET* and *CD44* genes corresponding to each individual case of gastric cancers and normal gastric tissues (circle). Real-time quantitative PCR was performed to evaluate their copy numbers in a cohort of gastric cancers and normal gastric tissues. Details are as described in Methods. Horizontal lines indicate a 95% confidence interval for the sample mean. T: tumor tissues; N: normal gastric tissues.

Real-time quantitative PCR assay was performed to analyze the copy number of thirty candidate genes in 30 pairs of gastric cancer and normal gastric tissues. With a gene copy number of 4 or more defined as gene amplification, we found that *ERBB4*, *C-MET* and *CD44* genes were frequently amplified in gastric cancers, however, other genes were not or infrequently amplified in gastric cancers, ranging from 0 to 8% (data not shown). Subsequently, we used the same method to analyze the copy number of *ERBB4*, *C-MET* and *CD44* genes in the 128 gastric cancers and 37 normal controls. The data showed that the prevalence of amplification of *ERBB4*, *C-MET* and *CD44* was 67% (86/128), 30% (39/128) and 66% (84/128), respectively, but not in the normal gastric tissues. Copy number of each gene corresponding to each individual case of gastric cancers and normal gastric tissues was showed in Figure 1. Statistical analysis showed that the copy number of each gene in gastric cancers was significantly higher than normal gastric tissues (Figure 1).

### 2.2. Association of Amplification of ERBB4, C-MET and CD44 with Clinicopathological Characteristics in Gastric Cancer

Because highly frequent amplification of *ERBB4*, *C-MET* and *CD44* was demonstrated in gastric cancer, their association with clinicopathological characteristics was analyzed in a cohort of clinically well-characterized gastric cancers. As shown in Table 1, there was a positive association of amplification of *ERBB4* (OR = 2.62, 95% CI = 1.23–5.59, *P* < 0.05) and *CD44* (OR = 2.28, 95% CI = 1.08–4.79, *P* < 0.05) with tumor differentiation. *C-MET* amplification was found to be significantly positively associated with tumor invasion (OR = 2.00, 95% CI = 1.03–3.89, *P* < 0.05). *CD44* amplification was significantly positively associated with lymph node metastasis (OR = 2.23, 95% CI = 1.05–4.73, *P* < 0.05). Moreover, our data also showed that *CD44* amplification was significantly positively associated with the number of lymph node metastasis (OR = 1.70, 95% CI = 1.08–2.69, *P* < 0.05). Notably, there was a significantly positive association of amplification of these genes with cancer-related death (Table 1). In order to assess the independent association of gene amplification with age, tumor differentiation, tumor stage, lymph node metastasis and survival status, we conducted multiple multivariable logistic regressions (Table 2). Similar to univariate analysis, after adjustment, amplification of *ERBB4* (OR = 2.95, 95% CI = 1.27–6.86, *P* < 0.05) and *CD44* (OR = 2.49, 95% CI = 1.08–5.79, *P* < 0.05) remained significantly associated with poor tumor differentiation (Table 2). Similarly, amplification of these genes remained significantly positively associated with cancer-related death (Table 2).

### 2.3. The Effect of Amplification of ERBB4, C-MET and CD44 on Poor Survival in Gastric Cancer

**Figure 2:**
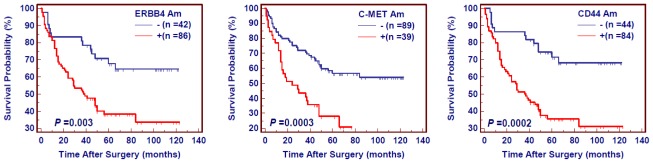
The effect of amplification of *ERBB4*, *C-MET* and *CD44* on poor survival in gastric cancer. Kaplan-Meier survival curves were made according to the presence of gene amplification in a cohort of gastric cancers. The patients with gene amplification had significantly shorter survival times than the patients without gene amplification. Am, amplification; +, harboring gene amplification; −, the lack of gene amplification.

The Kaplan-Meier estimator of the survivorship function was used to evaluate the impact of amplification of these three genes on the survival of gastric cancer patients. The survival of gastric cancer patients with and without gene amplification was compared using the log-rank test. As shown in Figure 2, amplification of *ERBB4*, *C-MET* and *CD44* significantly affected the poor survival of gastric cancer patients.

**Figure 3:**
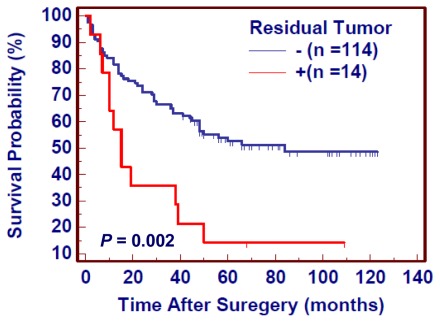
The effect of residual tumor after surgery on poor survival in gastric cancer. Survival was evaluated according to the presence of residual tumor after surgery in gastric cancers. Kaplan–Meier survival curves show that the patients with residual tumor had a significantly shorter survival time than the patients without residual tumor (*P* = 0.002). +, the patients with residual tumor; −, the patients without residual tumor.

**Figure 4:**
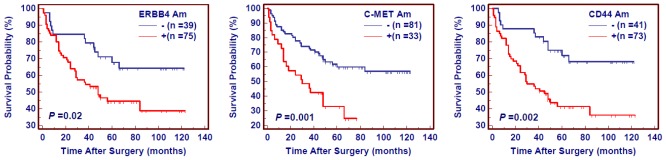
The effect of amplification of *ERBB4*, *C-MET* and *CD44* on poor survival of gastric cancer patients without residual tumor after surgery. Kaplan–Meier analysis of survival was performed according to the status of gene amplification in a cohort of gastric cancers. The patients with gene amplification had poorer survival than the patients without gene amplification. Am, amplification; +, harboring gene amplification; −, the lack of gene amplification.

Numerous evidences showed that residual tumor after surgery is an independent risk factor for gastric cancer patients. We thus attempted to evaluate the effect of residual tumor after surgery on the survival of gastric cancer patients. As shown in Figure 3, the patients with residual tumor after surgery had significantly shorter survival times than the patients without residual tumor (343.2 months *vs.* 601.2 months on average, *P* = 0.03). Given the impact of residual tumor after surgery on poor survival in gastric cancer, we excluded the patients with residual tumor to explore the effect of gene amplification on poor prognosis of gastric cancer patients. Similar to the findings in Figure 2, the patients with gene amplification had shorter survival times than the patients without gene amplification (*ERBB4*: 508.8 months *vs.* 777.6 on average, *P* = 0.002; *C-MET*: 382.8 months *vs.* 690.0 months on average, *P* = 0.0005; *CD44*: 490.8 months *vs.* 798.0 months on average, *P* = 0.0002) (Figure 4). Multivariate Cox regression analysis indicated that gene amplification may be served as a predictor of poor prognosis for gastric cancer patients (*ERBB4*: HR = 2.00, 95% CI = 1.02–3.86, *P* = 0.04; *C-MET*: HR = 2.10, 95% CI = 1.20–3.69, *P* = 0.01; *CD44*: HR = 2.59, 95% CI = 1.34–5.01, *P* = 0.005) as an independently variable with respect to gender, age, differentiation, lymph node metastasis, and tumor stage (Table 3).

**Figure 5:**
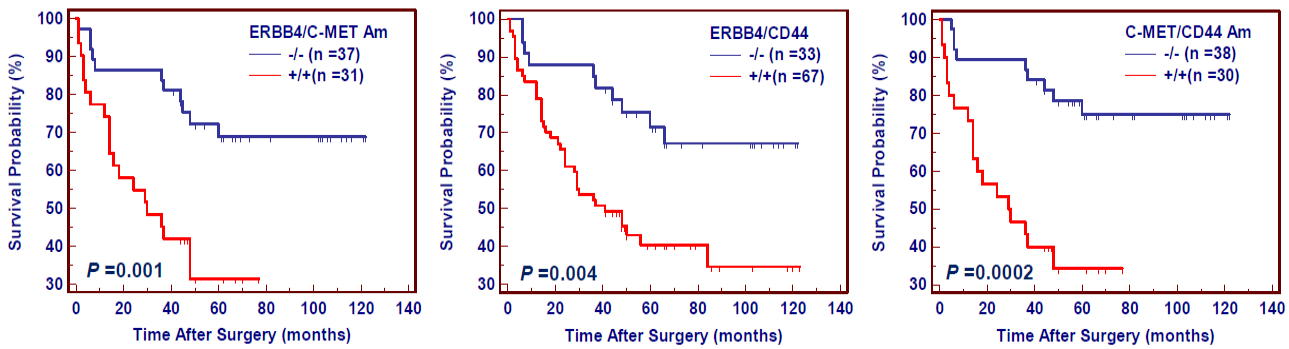
The effect of concomitant amplification of two of three genes on poor survival in gastric cancer. Survival was evaluated according to the presence of concomitant amplification of two of three genes in a number of gastric cancer patients without residual tumor after surgery. The patients with gene amplification had significantly shorter survival times than the patients without gene amplification. +/+, harboring concomitant amplification of two genes; −/−, the lack of gene amplification.

Gastric cancer is chronic proliferative disease characterized by genetic alterations of multiple genes, including gene amplification. In the present study, a high prevalence of concomitant amplification of 2 of 3 genes was found in gastric cancer, ranging from 28% (36/128) to 60% (77/128). As shown in Figure 5, concomitant amplification of 2 of 3 genes significantly shortened survival times (*ERBB4/C-MET*: 378.0 months *vs.* 795.6 on average, *P* = 0.001; *ERBB4/CD44*: 483.6 months *vs.* 817.2 months on average, *P* = 0.0004; *C-MET/CD44*: 372.0 months *vs.* 822.0 months on average, *P* < 0.0001), and might be more prognostic of poor survival than amplification of individual gene in gastric cancer.

## 3. Discussion

Although gastric cancer is a lethal disease around the world, the causes of gastric cancer are not completely understood. What is clear is that gastric cancer is a multistep process involving multiple genetic and epigenetic events, such as gene amplification. Gene amplification is frequent in solid tumors and represents one of the major molecular pathways through which the oncogenic potential of proto-oncogenes is overactivated during tumorigenesis [7,8]. Thus, gene amplification in general and specifically amplicons, have importance for both prognosis and targeted therapies in human cancers, including gastric cancer [9–12].

In the present study, we identified three frequently amplified genes in a cohort of well-characterized gastric cancer using real-time quantitative PCR method, including *ERBB4*, *C-MET* and *CD44*. ERBB4 (or HER4) is a member of the Tyr protein kinase family and the epidermal growth factor receptor subfamily, and plays an important role in normal cell growth and in neoplastic transformation [13]. *ERBB4* amplification, resulting in its overexpression and ligand-independent activation has been found in a variety of human malignancies [6,14]. *C-MET* oncogene encodes the receptor tyrosine kinase (RTK) for hepatocyte growth factor (HGF) and regulates genetic programs leading to cell growth, invasion and protection from apoptosis during tumorigenesis [15]. *C-MET* is frequently amplified in human cancers, including gastric cancer [16,17]. Its amplification is closely associated with poor prognosis of cancer patients and resistance to tyrosine kinase inhibitors [16–20]. CD44 is a receptor for hyaluronic acid (HA), which plays an important role in cell migration, tumor growth and progression through its affinity for HA, possibly also through its affinity for other ligands such as osteopontin, collagens, and matrix metalloproteinases (MMPs) [21]. Accumulating evidences have shown that CD44 is abundantly expressed in cancer-initiating cells (CICs), and has thus been implicated as a CIC marker in several malignancies of haematopoietic and epithelial origin, including gastric cancer [21,22]. Moreover, *CD44* amplification is also found in gastric cancer [6]. Notable, there was a higher amplification frequency of these genes in the present study than that of studies carried out in Europe and the US [23]. One possibility is that the different dietary and environmental factors, such as nitrates, carbohydrates and salt, may potentially cause these distinct genetic alterations in gastric carcinogenesis [24].

Given gene amplification plays a critical role in gastric tumorigenesis, we investigated their clinical significances and prognostic values in a cohort of gastric cancer patients who had known survival data. Our data showed that amplification of these genes was closely associated with poor tumor differentiation and cancer-related death, suggesting that these genes may contribute to oncologic outcomes of gastric cancer patients. More importantly, similar to observations in the previous studies [17], *C-MET* amplification was correlated with poor survival of gastric cancer patients in the present study. Notably, our data showed that amplification of *ERBB4* and *CD44* was significantly associated with poor prognosis in gastric cancer, is new to the literature.

To date, inhibitors and antibodies targeting specific molecules are vigorously being developed, and some have been demonstrated to be effective in clinical settings, including gastric cancer [25,26]. In the present study, we identified three frequently amplified genes and demonstrated that they were significantly associated with clinical outcome in gastric cancer, suggesting that they may be served as potential therapeutic targets for gastric cancer. Moreover, the prognostic markers may have another role in predicting and guiding the clinical treatment of cancer patients by allowing the identification of patients suited to current therapies. For example, *C-MET* amplification may identify a subset of epithelial cancers with extreme sensitivity to the selective tyrosine kinase inhibitor and define a patient group that is appropriate for clinical trials of targeted therapy using MET inhibitors [27].

## 4. Experimental Section

### 4.1. Patients

With the approval of our institutional review board and human ethics committee, where required, we studied 128 patients with gastric cancer who underwent surgery at the First Affiliated Hospital of Xi’an Jiaotong University School of Medicine from 1999 to 2006. A total of 37 tissues from the patients with chronic gastritis were obtained from the First Affiliated Hospital of Xi’an Jiaotong University School of Medicine as normal controls. None of these patients received chemotherapy and radiotherapy before the surgery. Informed consent was obtained from each patient before the surgery. The histologic diagnosis of tumors was made and agreed upon by at least two senior pathologists at Department of Pathology of the Hospital based on World Health Organization (WHO) criteria. Relevant clinicopathologic characteristics were obtained from the patients’ files or by interview with the patients or their relatives, and the details were summarized in Table 4.

### 4.2. Tissues and DNA Preparation

Serial sections from each tumor sample were cut. One section (5 μm) was stained by hematoxylin and eosin (H & E), and was marked as a tumor representative tissue by an expert surgical pathologist for gastric cancer. The next section (8 μm) was deparaffinized and stained with hematoxylin. Tumor tissues were isolated by manual microdissection under an inverted microscope using the marked H & E section for target tissue identification. Genomic DNA was extracted from isolated tissues as previously described [28]. Briefly, the tissues dissected were first treated with xylene for 12 hours at room temperature to remove the paraffin. All tissues were subsequently subjected to digestion with 1% sodium dodecyl sulfate (SDS) and proteinase K at 48 °C for 48 to 72 hours with addition of several spiking aliquots of concentrated proteinase K to facilitate digestion. Genomic DNA was isolated from the digested tissues followed by standard phenol-chloroform extraction and ethanol precipitation protocol, and stored at −80 °C until use.

### 4.3. Copy Number Analysis

We analyzed the copy number of candidate genes in gastric cancers and normal gastric tissues by real-time quantitative PCR technique on a CFX384 Thermal Cycler Dice^TM^ real-time PCR system (Bio-Rad Laboratories, Inc., Hercules, CA, USA) as described previously [29]. This method was well established and validated by florescence *in situ* hybridization (FISH) [29,30], which has been widely used in the various human cancers [28–32]. Specific primers and TaqMan probes were designed using Primer Express 3.0 (Applied Biosystems: Foster City, CA, USA, 2004) to amplify these genes and the internal reference gene *β-actin*. The primers and TaqMan probes for *ERBB4*, *C-MET*, *CD44* and *β-actin* genes in the present study were presented in Table 5. The primers and TaqMan probes for other 27 genes need be requested, including *SHFM1*, *CARD4*, *ELN*, *ARF5*, *SLC25A40*, *NRAS*, *CDK5*, *CREM*, *LMO2*, *DNMT1*, *FMR2*, *PSPHL*, *KRAS*, *PEG10*, *CDC2L5*, *HRAS*, *MGAM*, *ZP3*, *EPO*, *GUSB*, *ZPBP*, *TMEM60*, *PEPIN1*, *BRAF*, *AHR*, *DNMT3B* and *EZH2*. All TaqMan probes were labeled with 5′-FAM (6-carboxyfluorescein, fluorescent reporter) and 3′-TAMRA (6-carboxy-tetramethylrhodamine, fluorescent quencher). Using a PCR protocol described previously [29], PCR amplification were carried out in buffer containing 16.6 mM ammonium sulfate, 67 mM Tris base, 2.5 mM MgCl_2_, 10 mM 2-mercaptoethanol, 0.1% DMSO, 0.2 mM each of dATP, dCTP, dGTP and dTTP, 600 nM each of forward and reverse primers, 200 nM TaqMan probe, 0.6 unit Platinum *Taq* polymerase and 2% Rox reference dye. Each sample was run in triplicate, and *β-actin* was run in parallel to standardize the input DNA. Standard curves were established using serial dilutions of normal leukocyte DNA with a quantity range of 3.75 to 60 ng per 2 μL. The specificity of real-time quantitative PCR for these genes was confirmed by running thee PCR products on a 1.5% agarose gel to show single specific bands of the PCR products at the expected sizes (data not shown). The efficiency of real-time quantitative PCR assays for each target was shown in Table 5. Gene amplification was defined by a copy number ≥4.

### 4.4. Statistical Analysis

The Mann–Whitney *U* test was used to compare copy number of these genes between gastric cancer and normal gastric tissues. Association of gene amplification with clinicopathological characteristics was assessed univariately using the SPSS statistical package (version 11.5; IBM Corporation: Chicago, IL, USA, 2003). Multivariate models were then developed that adjusted for the most important covariates, including age, differentiation, tumor stage, lymph node metastasis and survival status. Survival length was determined from the day of primary tumor surgery to the day of death or last clinical follow-up. The Kaplan–Meier method was used for survival analysis grouping with gene amplification. Differences between curves were analyzed using the log-rank test. Multivariate Cox regression analysis was used to evaluate the effect of gene amplification on survival of independently of gender, age, differentiation, lymph node metastasis, and tumor stage. All statistical analyses were performed using the SPSS statistical package. *P* values < 0.05 were considered significant.

## 5. Conclusions

In summary, we identified three genes that were frequently amplified from thirty candidate genes in cancerous tissues, but not normal gastric tissues, and demonstrated that amplification of these genes was closely associated with clinicopathological characteristics, particularly tumor differentiation and cancer-related death. Importantly, amplification of these genes was significantly associated with poor survival in gastric cancer. Thus, these aberrantly amplified genes may be used as useful markers in evaluating poor prognosis and potential therapeutic targets for gastric cancer.

## Figures and Tables

**Table 1:** Amplification of individual genes in gastric cancer—univariate associations with clinicopathological characteristics (OR † and 95% CI).

Genes	Male *vs.* Female	Age 1	Tumor Localization 2	Tumor Size 3	Differentiation 4
***ERBB4***	0.97 (0.39–2.39)	1.12 (0.79–1.54)	0.82 (0.53–1.29)	0.88 (0.55–1.40)	2.62 (1.23–5.59) *
***C-MET***	0.95 (0.38–2.41)	1.29 (0.90–1.85)	0.92 (0.59–1.44)	1.28 (0.80–2.05)	1.92 (0.87–4.27)
***CD44***	1.06 (0.43–2.61)	1.15 (0.82–1.63)	0.80 (0.52–1.25)	0.88 (0.56–1.39)	2.28 (1.08–4.79) *

**Genes**	**Tumor Invasion**^5^	**Tumor Stage**^6^	**Lymph Node Metastasis**	**No. of LNM**^7^	**Survival Status**^8^

***ERBB4***	1.09 (0.65–1.85)	1.21 (0.81–1.83)	1.88 (0.89–4.01)	1.56 (0.99–2.46)	3.06 (1.41–6.63) **
***C-MET***	2.00 (1.03–3.89) *	1.33 (0.86–2.08)	1.81 (0.80–4.09)	1.25 (0.82–1.92)	3.42 (1.51–7.71) **
***CD44***	1.28 (0.76–2.13)	1.37 (0.91–2.06)	2.23 (1.05–4.73) *	1.70 (1.08–2.69) *	4.08 (1.86–8.94) **

† OR: odds ratio with 95% confidence interval;

1 Age (per 10 years);

2 Tumor localization (gastric cardia; gastric body; gastric antrum);

3 Tumor size (≤3 cm; >3 cm and ≤5 cm; >5 cm);

4 Differentiation (well or moderate; poor or no differentiation);

5 Tumor invasion (T1; T2; T3; T4);

6 Tumor stage (I; II; III; IV);

7 No. of LNM (lymph node metastasis) (0; 1–6; 7–15; >16);

8 Survival status (Alive *vs.* Dead);

* *P* < 0.05;

** *P* < 0.01.

**Table 2:** Amplification of individual genes in gastric cancer—multivariable models assessing age, differentiation, tumor stage, lymph node metastasis and survival status (OR † and 95% CI).

Genes	Age 1	Differentiation 2	Tumor Stage 3	Lymph Node Metastasis	Survival Status 4
***ERBB4***	1.22 (0.81–1.83)	2.95 (1.27–6.86) *	0.73 (0.39–1.39)	1.24 (0.38–3.98)	3.33 (1.28–8.67) *
***C-MET***	1.32 (0.88–1.97)	2.17 (0.92–5.14)	0.88 (0.45–1.70)	0.80 (0.23–2.84)	3.81 (1.35–10.8) *
***CD44***	1.19 (0.79–1.78)	2.49 (1.08–5.79) *	0.80 (0.43–1.51)	1.20 (0.38–3.84)	4.23 (1.62–11.0) **

† OR: odds ratio with 95% confidence interval;

1 Age (per 10 years);

2 Differentiation (well/moderate; poor/no differentiation);

3 Tumor stage (I; II; III; IV);

4 Survival status (Alive *vs.* Dead);

* *P* < 0.05;

** *P* < 0.01.

**Table 3:** The effect of amplification of *ERBB4*, *C-MET* and *CD44* on overall survival in gastric cancer using multivariate Cox regression analysis.

Covariate	Gene Amplification	*P* Value	HR *	95% CI
Gender	*ERBB4*	0.04	2.00	1.02–3.86
Age				
Differentiation	*C-MET*	0.01	2.10	1.20–3.69
Lymph node metastasis	*CD44*	0.005	2.59	1.34–5.01
Tumor stage				

* HR: Hazard ratio; CI: confidence interval; Significant at *P* < 0.05.

**Table 4:** Characteristics of patients with gastric cancer.

Characteristics	No. of Patients (%)
Gender	
Male	101 (78.9)
Female	27 (21.1)
Age, years	
Mean	59.42
SD	13.062
Tumor localization	
gastric cardia	35 (27.3)
gastric body	33 (25.8)
gastric antrum	60 (46.9)
Tumor size (cm^3^)	
≤3	43 (33.6)
3–5	46 (35.9)
>5	39 (30.5)
Differentiation	
well/moderate	53 (41.4)
poor/undifferentiation	75 (58.6)
Tumor invasion	
T1	14 (10.9)
T2	22 (17.2)
T3	90 (70.3)
T4	2 (1.6)
TNM stage	
I	29 (22.7)
II	20 (15.6)
III	73 (57.0)
IV	6 (4.7)
Residual tumor	
Yes	14 (10.9)
No	114 (89.1)
Lymph node metastasis (LNM)	
Yes	80 (62.5)
No	48 (37.5)
No. of LNM	
N0	48(37.5)
N1 (1–6)	47 (36.7)
N2 (7–15)	27 (21.1)
N3 (≥16)	6 (4.7)
Survival status	
Dead	66 (51.6)
Alive	62 (48.4)

**Table 5:** The primer and TaqMan probe sequences used in this study.

Genes	Forward Primer Sequence (5′→3′)	Probe Sequence (5′→3′)	Reverse Primer Sequence (5′→3′)	Amplification Efficiency (%)
***ERBB4***	CCCTGAAGCCAGGCACTGT	6FAM-CTGCCGCCTCCACCTTACAGACACC-TAMRA	CCTAAAAAACCACAACTGAGCTTACA	84.2
***C-MET***	ACCTGCCAGCGACATGTCTT	6FAM-CCACAATCATACTGCTGACA-TAMRA	GACACTGGCTGGGCTCTTCTATC	84.1
***CD44***	GCTCTGAGCATCGGATTTGAG	6FAM-CCTGCAGGTAAGAGACCAGCACCCG-TAMRA	AGGCCGCCAGCTTTCC	85.0
***β-Actin***	TCACCCACACTGTGCCCATCTACGA	6FAM-ATGCCCTCCCCCATGCCATCC-TAMRA	TCGGTGAGGATCTTCATGAGGTA	95.0
